# Long-term disturbance dynamics and resilience of tropical peat swamp forests

**DOI:** 10.1111/1365-2745.12329

**Published:** 2015-01-07

**Authors:** Lydia E S Cole, Shonil A Bhagwat, Katherine J Willis

**Affiliations:** 1 Oxford Long-term Ecology Laboratory, Department of Zoology, University of OxfordSouth Parks Road, Oxford, OX1 3PS, UK; 2 Biodiversity Institute, Oxford Martin School, Department of Zoology, University of OxfordSouth Parks Road, Oxford, OX1 3PS, UK; 3 Department of Geography, Faculty of Social Sciences, The Open UniversityWalton Hall, Milton Keynes, MK7 6AA, UK; 4 Department of Biology, University of BergenP.O. Box 7803, N-5020, Bergen, Norway; 5 Royal Botanical GardensKew, Richmond, Surrey, TW9 3AB, UK

**Keywords:** climate change, El Niño, fire, fossil pollen, land-use history, palaeoecology, Sarawak, sustainable management, tropical wetlands, vegetation change

## Abstract

**1.** The coastal peat swamp forests of Sarawak, Malaysian Borneo, are undergoing rapid conversion, predominantly into oil palm plantations. This wetland ecosystem is assumed to have experienced insignificant disturbance in the past, persisting under a single ecologically-stable regime. However, there is limited knowledge of the past disturbance regime, long-term functioning and fundamentally the resilience of this ecosystem to changing natural and anthropogenic perturbations through time.

**2.** In this study, long-term ecological data sets from three degraded peatlands in Sarawak were collected to shed light on peat swamp forest dynamics. Fossil pollen and charcoal were counted in each sedimentary sequence to reconstruct vegetation and investigate responses to past environmental disturbance, both natural and anthropogenic.

**3.** Results demonstrate that peat swamp forest taxa have dominated these vegetation profiles throughout the last *c*. 2000-year period despite the presence of various drivers of disturbance. Evidence for episodes of climatic variability, predominantly linked to ENSO events, and wildfires is present throughout. However, in the last *c*. 500 years, burning and indicators of human disturbance have elevated beyond past levels at these sites, concurrent with a reduction in peat swamp forest pollen.

**4.** Two key insights have been gained through this palaeoecological analysis: (i) peat swamp forest vegetation has demonstrated resilience to disturbance caused by burning and climatic variability in Sarawak in the late Holocene, however (ii) coincident with increased fire combined with human impact *c*. 500 years ago, these communities started to decline.

**5.** *Synthesis*. Sarawak's coastal peat swamps have demonstrated resilience to past natural disturbances, with forest vegetation persisting through episodes of fire and climatic variability. However, palaeoecological data presented here suggest that recent, anthropogenic disturbances are of a greater magnitude, causing the observed decline in the peat swamp forest communities in the last *c*. 500 years and challenging the ecosystem's persistence. This study greatly extends our knowledge of the ecological functioning of these understudied ecosystems, providing baseline information on the past vegetation and its response to disturbance. This understanding is central to developing management strategies that foster resilience in the remaining peat swamp forests and ensure continued provision of services, namely carbon storage, from this globally important ecosystem.

## Introduction

Southeast Asia's peat swamp forests are globally important ecosystems, storing approximately 12% of the World's peatland carbon below-ground (Page, Rieley & Banks 2011) as well as supporting a wide diversity of floral, faunal and human communities above the surface (Silvius & Giesen 1996; Ewel 2010; Yule 2010). Despite these noteworthy characteristics, relatively little is known about how these ecosystems have changed through time, and how resilient they are to natural and anthropogenic disturbances. For example, reports are rife on the impacts of recent fire disturbance on peatlands and the carbon they store (e.g. Page *et al*. 2002; CIFOR 2014). Although fire is a natural and important part of many forest ecosystems (Pausas & Keeley 2009), widespread and frequent fires are potentially challenging the resilience of these peat swamp forests. The classical definition of resilience, adopted here, is ‘the ability of an ecosystem to maintain its structure and function despite disturbance’ (Holling 1973), though definitions and measurement methods are numerous (Ludwig, Walker & Holling 1997; Carpenter *et al*. 2001). A loss of resilience occurs when an ecosystem is perturbed beyond its threshold by extreme physical or biological conditions, causing a change in its ecological composition and functioning (Ficetola & Denoël 2009; Bhagwat, Nogué & Willis 2012) to such a degree that a return to its former state is prohibited (Scheffer & Carpenter 2003). Understanding the thresholds and resilience of these ‘fragile’ peat swamp forests in the face of burning, amongst other drivers of disturbance, represents a large knowledge gap.

The paucity of ecological information is especially apparent for the peatlands of Sarawak in Northern Borneo (Liong & Siong 1979), where over 80% of Malaysia's peat swamp forests are found (Page *et al*. 1999). Insights into their longevity and dynamics through time are provided by a limited number of studies (e.g. Anderson 1964; Anderson & Muller 1975; Morley 1981; Yulianto *et al*. 2005).

The coastal peat swamps of Sarawak provide a variety of resources, such as the economically valuable Dipterocarp *Shorea albida* (IUCN 1996) and non-timber forest products: fish, fruit and sago palm *Metroxylon sagu* to name a few. They also provide a range of ecosystem services: locally, they act as a buffer against flooding and drought (Andriesse 1988), prevent saline water intrusion (Liong & Siong 1979; Phillips 1998), as well as other more indirect services for communities (Silvius & Giesen 1996; Cole 2013); and globally, they represent a vast CO_2_ sink, playing a central role in strategies to mitigate carbon emissions (Page, Rieley & Banks 2011). It is widely reported that this is a vulnerable ecosystem (Page *et al*. 2004; UNFCCC 2013) and activities that disrupt the tight interrelationship between peat, water and vegetation (Dommain, Couwenberg & Joosten 2010), notably drainage (Hooijer *et al*. 2011), could challenge the resilience of the system, causing it to shift from a vast sink to an extensive source of greenhouse gases (Fargione *et al*. 2008; Ramdani & Hino 2013; Dommain *et al*. 2014). Changes in both future climate, especially reduced regional precipitation (Li *et al*. 2007), and land-use may also contribute to such a shift.

In Sarawak particularly, peat swamp forests are experiencing deforestation rates up to 12 times greater than those across Asia (SarVision 2011), and approximately 25% higher than those in the island's lowland dipterocarp forest (Langner, Miettinen & Siegert 2007). Peatlands are described as the final frontier for agricultural expansion (Koh *et al*. 2011). Most recently, this has been driven by the pulpwood industry and the rapid growth of the palm oil market (Murdiyarso, Hergoualc'H & Verchot 2010; Miettinen *et al*. 2012), as well as State development initiatives, for example, Sarawak Corridor of Renewable Energy (SCORE) (RCDA 2012). Prior to these recent developments, evidence suggests that people did not practice shifting cultivation or permanently settle in these ecosystems (Verhagen *et al*. 2004), mostly due to the waterlogged nature of the landscape (IUCN 1996), despite the extensive history of human presence and environmental exploitation in other parts of Borneo (Flenley 1988; Anshari *et al*. 2004; Hunt & Rushworth 2005; Yulianto *et al*. 2005; Hunt & Premathilake 2012).

In the face of today's high rates of tropical peat swamp forest conversion, it is important to understand the magnitude and impact of this disturbance relative to those experienced by the ecosystem in the past. Knowledge of the major forms of past disturbance and patterns of response of the peat swamp forest communities would provide information that could be used to inform the management of these areas today and in the modelling of their future carbon storage potential.

This study takes a palaeoecological approach to examine the disturbance dynamics of Sarawak's coastal peat swamps. Palaeoecology utilizes proxies, such as fossil pollen and micro- and macrofossil charcoal, to extend the scope of ecological studies to past ecosystems (Rull 2010; Willis *et al*. 2010). Accumulating peat is an excellent repository of such proxies, storing information on local environmental and climatic changes through time (Barber 1993; Zhao, Holzer & Zicheng 2007; Zhu *et al*. 2010). It also provides insights into patterns of human-induced disturbance, proving especially useful in these ecosystems which lack surface archaeology (Hunt & Rushworth 2005).

The aim of this study was to investigate the impact that different past disturbances have had on the dynamics of these highly threatened wetland ecosystems. In particular, we ask: (i) How has the vegetation of these peat swamp forests changed through time? (ii) What indicators of past disturbance are there and when? (with a focus on fire, climatic and human drivers) and (iii) How did the peat swamp forest vegetation respond to these disturbances? Placing contemporary levels of disturbance and vegetation change within the long-term context presented here allows us to inform how this ecosystem may respond to future global change (Haberle *et al*. 2010).

## Materials and methods

### Modern environmental setting

**Fig. 1:**
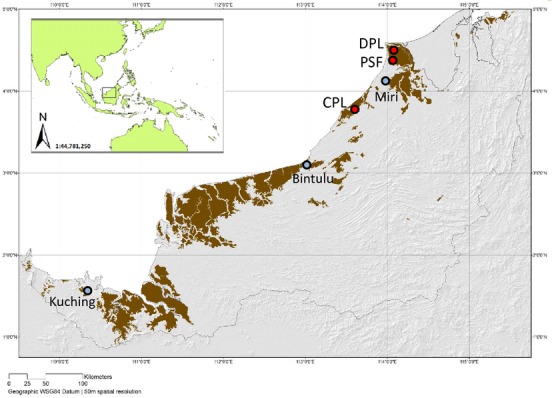
Map showing the geographical location of Sarawak, Malaysian Borneo (inner box), within Southeast Asia, annotated with the main settlements (blue circles) and three peat swamp sites (red circles) from which cores were extracted: DPL (Deforested Peatland), PSF (Peat Swamp Fragment) and CPL (Converted Peatland). [On the main map, peatland areas are represented by dark shading and the Sarawak State boundary by a grey line. Image courtesy of SarVision (2011)].

The peat swamp forests of Sarawak, one of the two East Malaysian States in northern Borneo (Fig.1), are predominantly found along the coast, covering approximately 13% of the State's land area (Wetlands International 2010). Borneo's tropical ever-wet climate (Morley & Flenley 1987; Sawal 2003) is important for the development of peat (Staub & Gastaldo 2003): a soil that comprises ≥ 65% organic matter (USDA 1975) is ≥ 50 cm in depth and at least one hectare in area (Liong & Siong 1979), though definitions differ (Page, Banks & Rieley 2010). The tight interrelationship between peat accumulation, forest vegetation and hydrological conditions (Dommain, Couwenberg & Joosten 2010; Posa, Wijedasa & Corlett 2011) makes this ecosystem more vulnerable to disturbance caused by deforestation than other forest types. Peat swamp forests house a range of species, capable of tolerating the high acidity, low nutrient availability and waterlogged nature of this habitat (Ewel 2010; Posa, Wijedasa & Corlett 2011).

### Data collection

Three sets of sediment cores, each comprising a series of continuous overlapping sequences, were extracted using a Russian corer in October 2009, from the interior of three peat areas across the Miri and Batu Niah Districts of north-east Sarawak: Deforested Peatland from Senadin, Kuala Baram; Peat Swamp Fragment from Sungai Dua Forest Reserve; and Converted Peatland from Sungai Niah (Table 1). Core sediment sections of 50 cm were recovered until the majority of material extracted was no longer peat. Sections were then wrapped in thin plastic film and tin foil and kept out of light and below 5 °C where possible, to prevent sample contamination, drying or decomposition and promote pollen preservation. All materials were transported back to the Long-Term Ecology Laboratory at Oxford University, UK, for analysis.

**Table 1:** Details of the coring sites and basic core attributes

Site I.D.	Site name	Lat. long.	Elevation (m)	Land-use type	Vegetation type	Length of core (cm)	No. sub-samples
DPL	Deforested peatland	04°30′47″ N, 114°2′47″ E (4.513056, 114.046389)	11	Large area of fire-prone semi-drained peatland	Open, Cyperaceae and fern dominated	285	33
PSF	Peat swamp fragment	04°21′24″ N, 114°0′21″ E (4.356667, 114.005833)	17	Small patch of peat swamp forest on outskirts of Miri	Closed, peat swamp tree dominated	382	52
CPL	Converted peatland	03°52′4″ N, 113°42′43″ E (3.867778, 113.711944)	6	Fallow land adjacent to small paddy plot and oil palm (*Elaeis guineensis*) plantation	Open with small forest patches, herb and grass dominated	318	61

### Palaeoecological analysis

For each core sequence, the sediment stratigraphy was first described according to the tripartite Troels-Smith classification system (i.e. composition, degree of humification and physical properties) (Troels-Smith 1955), prior to combining the separate elements into a summary scheme. This was then graphically displayed alongside the palaeoecological data, to provide additional evidence for major sedimentation changes (Yeloff *et al*. 2006), associated with, for example, a mangrove to peat ecosystem transition. Using standard techniques (Bennett & Willis 2001), fossil pollen was extracted from each sediment core at regular intervals (see Appendix S1 in Supporting Information). A known concentration of ‘marker’ spores of the clubmoss genus, *Lycopodium*, was added, in the form of two tablets (prepared at the University of Lund, Batch No. 1031), to each sample in order to determine pollen concentration in each 1 cm^3^ of extracted sediment. *Lycopodium* is exotic to these peatland ecosystems and thus by counting the number of spores that arise simultaneously with the fossil pollen count for each sample, a ratio of ‘marker’ spores to the original concentration added can be calculated and used to quantify the concentration of pollen grains per cm^3^. A minimum of 300 pollen grains were counted per sampling level using a Meiji microscope, at 400× magnification. Indeterminate pollen (i.e. grains that were deformed, obscured or unidentified) was excluded, in addition to pollen from Poaceae and Cyperaceae and fern spores (primarily of monolete form), which can be disproportionately abundant and thus obscure vegetation interpretations (e.g. see Bush 2002). Microcharcoal was counted simultaneously on the pollen slides, according to Clark's point count method (Clark 1982), and macrocharcoal content was determined using a light microscope, at the same intervals as fossil pollen, in order to broadly reconstruct regional and local fire events, respectively (Clark 1988).

**Fig. 2:**
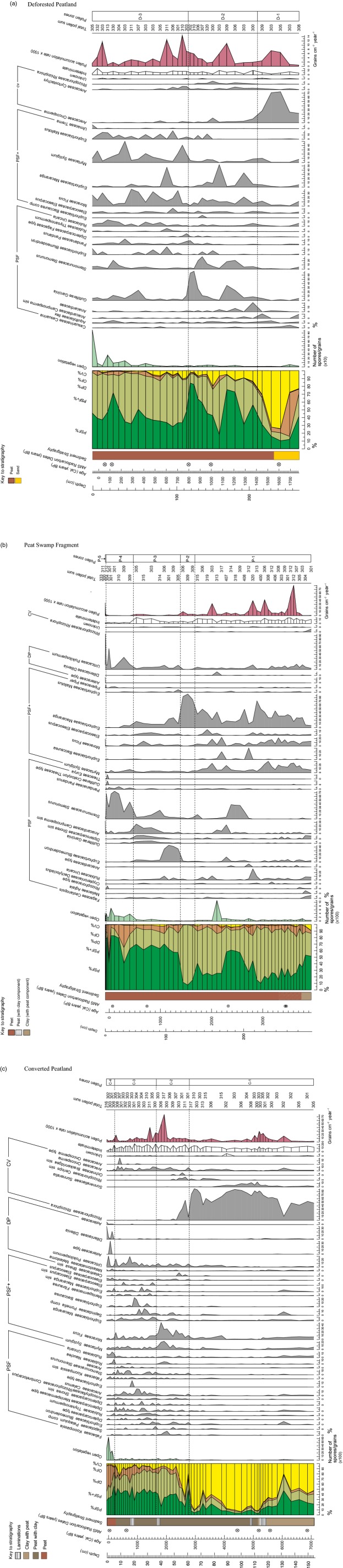
Summary pollen diagrams, displaying the pollen sum (far left), selected pollen taxa and key indicators of ecological change for each site: (a) Deforested Peatland, (b) Peat Swamp Fragment and (c) Converted Peatland. Only pollen taxa that contribute > 5% to the pollen sum, at any one level, are included. Ecological group classifications, in order of their position in the pollen sum, are as follows (full descriptions are in Table 2): PSF – peat swamp forest; PSF+ – peat swamp forest pioneers; DP – degraded peat; OF – other forest, and CV – coastal vegetation. (OF vegetation contributes < 5% to the pollen sum in all three sites.) The system of notation adopted for reflecting the certainty of taxonomic identification follows that of Benninghoff & Kapp (1962): ‘comp’ indicates a grain that is almost certainly the same as the reference taxon; ‘sim’, one that is more similar to the reference taxon than any other known reference taxa, but there is less certainty in the association; and ‘type’, a grain corresponds with one morphology within a polymorphic taxonomic unit.

Reference collections for pollen identification were gathered from Queen's University Belfast, The Royal Botanical Gardens in Kew, the Plant Sciences Department in Oxford, and from within Oxford University's Long-Term Ecology Laboratory. The Pollen Flora of Taiwan (Huang 1972), and plates found in Stuijts (1993) and Anderson & Muller (1975) were also used for identification of grains. Due to the diversity of species in the peat swamp flora and differing levels of pollen production, taxa identified through pollen counting were allocated to ecological groups (Table 2) to aid interpretation of the palaeo-plant communities (e.g. Muller 1963), using the Checklist of Coode *et al*. (1996), as well as various other publications from the region (Anderson 1964; Anderson 1980; Stuijts 1993; Anshari, Kershaw & van der Kaars 2001; Anshari *et al*. 2004). The identification of the majority of plant taxa was not resolved beyond generic level, as is common amongst palaeoecological studies in tropical regions (e.g. Muller 1963; Anshari *et al*. 2004). Thus, a system of notation developed by Benninghoff & Kapp (1962) has been used to reflect the level of certainty in the identifications made (Fig.2). (See Appendix S2 in Supporting Information for plates of the common pollen grains and spore types recorded in this study.)

**Table 2:** Definition of ecological groups, acronyms used, and key indicator taxa, identified through fossil pollen analysis and used to reconstruct past vegetation dynamics (for a complete list of fossil pollen grains and spores counted, see Table S2 in Supporting Information; and for authorities on species listed in this manuscript, refer to Coode *et al*. (1996) and other publications referenced for ecological group classification)

Ecological group	Name	Explanation	Major plant taxa
PSF	Peat swamp forest	Mature taxa of peat swamp forest, assumed to grow in old-growth forest	*Combretocarpus* (Anisophyllaceae), *Shorea* (Dipterocarpaceae), *Stemonurus* (Stemonuraceae)
PSF+	Peat swamp forest – pioneers	Pioneer taxa of peat swamp forest, indicating an early successional plant community	*Elaeocarpus* (Elaeocarpaceae), *Macaranga* (Euphorbiaceae), *Ficus* (Moraceae)
DP	Degraded peat	Taxa not found in older-growth peat swamp forest or in greater abundance in disturbed areas of peat where the vegetation is open	*Dillenia* (Dilleniaceae), *Poikilospermum* (Urticaceae)
CV	Coastal vegetation	Coastal vegetation associated with succession to peat from mangrove/littoral habitat types	*Oncosperma* (Arecaceae), *Sonnneratia* (Sonneratiaceae)
OF	Other forest	Other forest (non-peat swamp forest) taxa, for example swamp forest or forest on mineral soils	*Terminalia* (Combretaceae), *Rubus* (Rosaceae)
OP	Open vegetation	Disturbance tolerant vegetation indicative of open environments greater than tree-fall gaps, not included in pollen sum	Monoletes, Triletes, Poaceae, Cyperaceae

Bulk sediment samples were sent for accelerator mass spectrometry (AMS) radiocarbon dating to either the ^14^Chrono Centre in the Archaeology and Palaeoecology Department at Queen's University Belfast, or the Scottish Universities Environmental Research Centre (SUERC) AMS Laboratory, after preparation to graphite at the Natural Environment Research Council (NERC) Radiocarbon Facility. The coding package *Clam* (Blaauw 2010), in R (R Core Team 2012), with a Northern Hemisphere correction (the IntCal04 curve), was used to calibrate the conventional radiocarbon dates (Table 3) and construct the best-fitting age-depth models (Appendix S3).

**Table 3:** Accelerator mass spectrometry (AMS) ^14^C ages along with calibrated ages for radiocarbon-dated samples from each core, calculated individually using Calib 601 (Stuiver, Reimer & Reimer 2005) (an age of 50 Cal. years BP was given to the PSF sample taken at 30 cm depth, to approximate the *modern* date reported for this sample after radiocarbon dating)

Lab code	Core	Depth (cm)	^14^C years BP (±1ơ)	δ^13^C (‰)	Calibrated years BP (±1ơ)	Dated material
SUERC-35243	CPL	30	618 ± 35	−30.2	603 ± 56	Soil
UBA-14322	CPL	97	4018 ± 26	−32.8	4475 ± 54	Soil
SUERC-35244	CPL	170	4486 ± 35	−29.4	5166 ± 129	Soil
SUERC-35245	CPL	225	4884 ± 37	−29.3	5623.5 ± 40.5	Soil
UBA-15751	CPL	300	6038 ± 32	−29.7	6880.5 ± 89.5	Soil
SUERC-35240	PSF	30	Modern	−29.9	50	Peat
UBA-15749	PSF	100	841 ± 25	−32.4	741.5 ± 48.5	Peat
SUERC-35241	PSF	155	2275 ± 37	−30.2	2209.5 ± 52.5	Peat
SUERC-35242	PSF	200	3270 ± 35	−30.7	3506 ± 70	Peat
UBA-15129	PSF	240	3242 ± 22	−31.6	3441 ± 46	Peat
SUERC-35235	DPL	60	119 ± 37	−29.5	80 ± 70	Peat
UBA-15750	DPL	100	797 ± 25	−29.3	708.5 ± 33.5	Peat
SUERC-35236	DPL	180	998 ± 37	−29.2	931 ± 40	Peat
UBA-15131	DPL	220	1602 ± 25	−26.4	1477 ± 63	Peat
SUERC-35239	DPL	260	1260 ± 35	−29.1	1201.5 ± 80.5	Peat

**Fig. 3:**
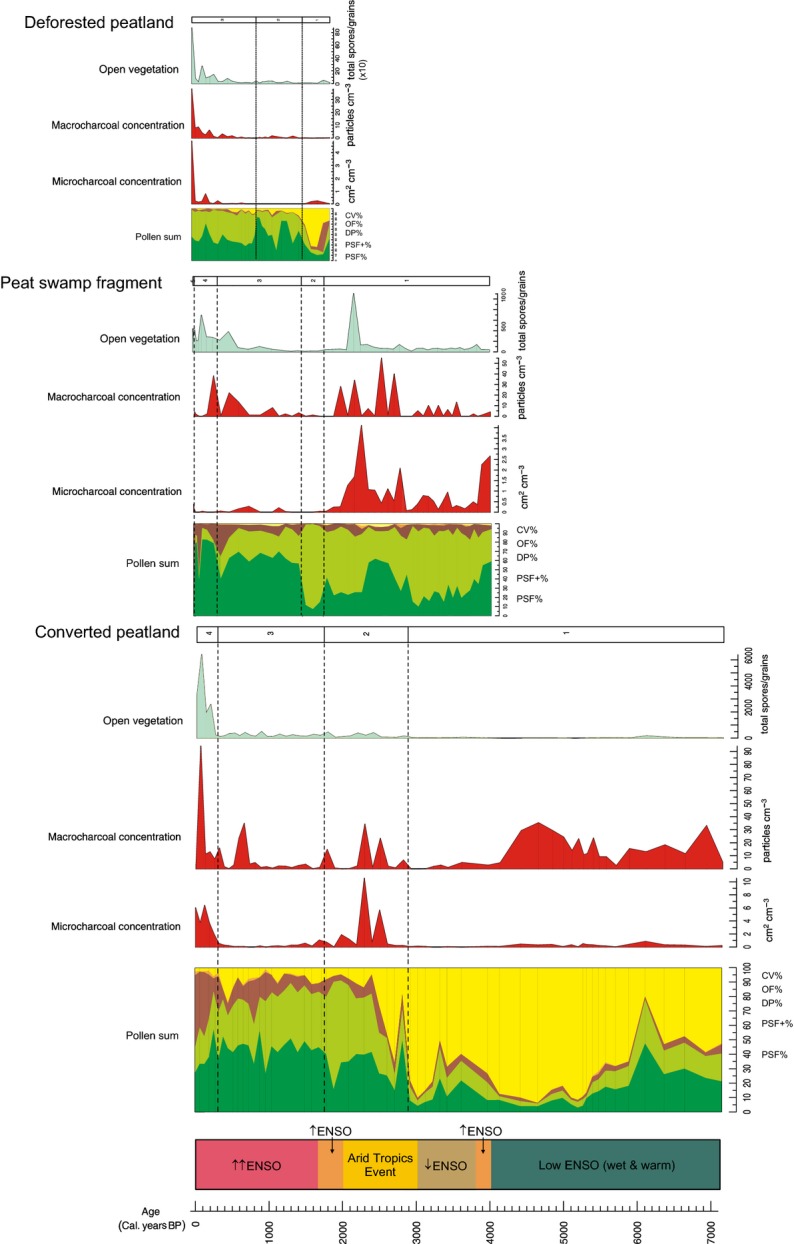
Main disturbance proxies and pollen sums for each site plotted against time. The approximate timing of major periods of climatic change, linked to the ENSO phenomenon, are marked (the direction and number of arrows describe the relative intensity of ENSO in comparison with its activity pre-5000 Cal. years BP) (for details of periods, see Table S1). Macro- and microcharcoal data represent past fire episodes and open vegetation taxa, a proxy for degraded forest and open areas linked to anthropogenic disturbance. The data sets of each core were adjusted to enable their chronological correspondence against one timescale. Significant pollen zones are shown for each (see Fig.2 for site-specific labelling, i.e. D-, P- and C-notation).

Relative abundance of pollen taxa was calculated using a total pollen sum, from which Poaceae and Cyperaceae grains and fern spores were excluded for aforementioned reasons. Instead, the counts of these three taxa, known to dominate canopy-free sites, were summed to form the ‘open vegetation’ group, providing an indicator for human disturbance. Cyperaceae and ferns are especially common in tropical peatlands (Flenley & Butler 2001), where their abundance indicates anthropogenic peat swamp forest degradation (van Eijk *et al*. 2009; Page *et al*. 2009). Significant pollen assemblage zones were constructed using an optimal splitting by information content technique on all data included in the pollen sum, after assessing the number of zones that were significant via a broken stick modelling approach across different data analyses (Bennett 1996). *psimpoll* version 4.26 (Bennett 1994) was used to display all pollen, spore and charcoal counts and disturbance proxies (Figs2 and 3).

Three key variables have been used in this study to indicate disturbance in these forests through time: charcoal counts (both macro- and microcharcoal) for fire disturbance, ‘open vegetation’ counts for human disturbance, and published regional climate records for climatic variability. Independently sourced climate data that can be used to investigate climate–vegetation relationships in the area of interest are limited, and those which are available fragmented (Partin *et al*. 2007) or from other regions (e.g. Grieβinger *et al*. 2011; Selvaraj *et al*. 2012). Thus, several different published records were used here to investigate the impact of precipitation (Grieβinger *et al*. 2011) and temperature changes (e.g. Mayewski *et al*. 2004; Partin *et al*. 2007; Selvaraj *et al*. 2011, 2012) on peat swamp vegetation, specifically focusing on climatic variability related to the El Niño Southern Oscillation (ENSO) (Fig.3). (See Table S1 in Supporting Information for a summarized description of climatic variability over the latter half of the Holocene and the list of references from which it was compiled.)

## Results

### Radiocarbon dates and stratigraphy

Radiocarbon dates were obtained for each sediment core (Table 3), providing an age-depth profile and allowing for comparison of disturbance events across these sites (Fig.3). Basal dates for Deforested Peatland and Peat Swamp Fragment show age inversions and therefore interpretation of pollen data recorded from these cores beyond 200 cm depth, equating to *c*. 1200 Cal. years BP and 3000 Cal. years BP, respectively, is tentative. Deforested Peatland covers the shortest time period, with the peat swamp starting to develop < 1500 Cal. years BP (Zone D-2, Fig.2a) on a silty-sandy substrate, suggestive of a riverine environment in proximity to the coast. In Peat Swamp Fragment, the peat swamp was present from *c*. 3500 Cal. years BP, developing on a clay substrate, probably also associated with a riverine environment. Converted Peatland shows a different pattern of development, with organic-rich deposits originating on what was predominantly a clay substrate *c*. 5000 Cal. years BP. After this point, a clay-peat soil started to accumulate, interspersed with laminations, which, coupled with the presence of coastal vegetation, indicates the existence of a tidally influenced estuarine mangrove habitat. Peat swamp forest did not start to develop in this site until *c*. 2800 Cal. years BP (Zone C-2, Fig.2c). Accumulation rates broadly reflect this transition in depositional environment and associated vegetation through time.

### Description of pollen diagrams

The majority of the 179 pollen types identified was from the PSF and PSF+ ecological groups, demonstrating that peat swamp forest has dominated in all three sites, with no major shifts in vegetation communities, since peat accumulation began (Fig.2). The other two ecological groups that appear most frequently in pollen counts are those comprising degraded peat and coastal vegetation (Fig.2). Several pollen taxa were common across sites, for example, *Dillenia* and *Poikilospermum*, common disturbance indicators associated with degraded peat, and *Oncosperma*, found in saline–freshwater transition zones. Approximately, 10 pollen types were not identified; levels of damaged or obscured grains and spores were greater and varied across samples and sites. The apparently random occurrence of unknown and indeterminate grains across the three sites through time does not have implications for the interpretation of this study's results. There was little concurrence of pollen concentration peaks across sites, except where concentrations broadly increase at the point of peat swamp forest development.

Although there does appear to be a shared pool of species that feature to some extent across all cores, there are unique peat swamp forest pollen assemblages within each site, with varying taxa and abundances through time and space, as exemplified by the different location of vegetation zones in most cases (Fig.3).

Despite the reported differences, there are three notable similarities observed across the three cores. One is the dominance of PSF vegetation through time, post-initiation of peat swamp development. The next is the strong presence of PSF+ taxa within the peat swamp forest, and frequent fluctuations between pioneer and mature taxa coinciding broadly in each site with changes in fossil charcoal levels. The final similarity is the sharp increase in open vegetation taxa across all sites within the last 1000 years, which, although at varying times, coincides with a change in vegetation zone in each (Fig.2). From *c*. 300 Cal. years BP, there is a particularly sharp increase, corresponding with the largest counts of degraded peat taxa in Converted Peatland and Peat Swamp Fragment sites.

### Change in disturbance indicators through time

Fire, human impact (inferred from large increases in open vegetation counts) and climatic change are the three disturbance types examined in this study (Fig.3).

Although to varying degrees, there is evidence for the presence of fire in all sites through time. In addition, there is a general coherence between micro- and macrocharcoal levels, signifying a degree of synchrony between local and regional fire events. One obvious exception, however, is the large quantity of macrocharcoal coinciding with low levels of microcharcoal from *c*. 7000 to 4000 Cal. years BP in the Converted Peatland site (Fig.3), indicating intense local burning, albeit in a different ecological context (Fig.2c). These high macrocharcoal levels are only exceeded here in the last 100 years. The Peat Swamp Forest site experienced greatest levels of local and regional fire between *c*. 2000 and 3000 Cal. years BP, after which microcharcoal declined significantly until the present day. Charcoal counts from the Converted Peatland site share this trend of heightened burning during this approximately 1000-year period, coincident with an arid episode in the Tropics (Selvaraj *et al*. 2011, 2012). The record for the Deforested Peatland site does not cover this period in time. Here, the highest levels of burning occur within the last *c*. 200 years. This pattern of increasing fire in the recent past is also seen in Converted Peatland and Peat Swamp Fragment sites, starting from *c*. 300 and 500 Cal. years BP, respectively.

The open vegetation count, after maintaining near-zero levels through the majority of the past in all sites, rises significantly after *c*. 500 Cal. years BP. This trend broadly follows that of charcoal in the latter half of the last millennium, but with a particularly dramatic increase within the last two centuries. Only in the Peat Swamp Fragment site was there a peak *c*. 2200 Cal. years BP (Fig.3).

The schematic summarizing variation in ENSO over the last 7000 years (Fig.3) demonstrates that there were notable changes in regional climate throughout the Holocene. However, comparison of the timings of climatic variability with each vegetation profile suggests that the ENSO phenomenon had little impact on peat swamp forest dynamics across these sites.

### Vegetation response to disturbance

Changes in pollen counts coincident with elevated charcoal levels do not show a clearly coherent signal across sites, though there are some notable patterns. During the period of elevated burning between *c*. 2000 and 3000 Cal. years BP in Peat Swamp Fragment and Converted Peatland (zones P-1 and C-2 respectively, Figs2 and 3), aligning with the Arid Tropics Events, PSF+ taxa increase. Conversely, in the approximately 1000-year period of greatly reduced fossil charcoal that follows, the count of mature PSF taxa relative to pioneers increases. During the last several hundred years of elevated burning across sites, the notable vegetation change is in the degraded peat (DP) taxa, which contributes a greater proportion to the pollen sum than observed throughout the recorded past in Peat Swamp Fragment and Converted Peatland sites, coincident with a reduced contribution by PSF and PSF+ taxa (zones P-4 and P-5, and C-4, respectively, Figs2 and 3).

The pattern of elevated DP counts relative to PSF vegetation types in two of the sites also coincides with the notable increases in open vegetation counts (an indicator for human disturbance) within the last several hundred years. A slight increase in DP taxa, and increased fluctuations across ecological groups, is visible in the Converted Peatland site during the last *c*. 1000 years in parallel with low, yet greater levels of open vegetation in the landscape. A similar response in DP% does not co-occur with the large spike in open vegetation observed in the Peat Swamp Fragment site *c*. 2200 Cal. years BP, though a notable increase in the relative proportion of PSF+ taxa appears to follow it. Deforested Peatland does not demonstrate the same trend in vegetation change with charcoal or open vegetation counts in the recent past.

In terms of climatic changes, there appear to be no coherent or notable vegetation responses across sites. However, there is a lack of information for each core on the baseline vegetation pre-ENSO intensification, which could be used to assess the impact of this climatic phenomenon on the peat swamp forest community.

## Discussion

This study characterizes the vegetation change in three coastal peat swamp forests in Sarawak over the late Holocene and the associated disturbance dynamics. It specifically identifies the past disturbance regimes in these ecosystems, focusing on evidence for fire, climatic or human perturbation, and explores how the peat swamp forest vegetation responded to these drivers. Using these insights, the impact of disturbance over time on the resilience of these ecosystems is considered.

### How has the vegetation of these peat swamp forests changed through time?

In each site post-peat swamp development, the baseline vegetation has comprised PSF, fluctuating at approximately 80% of the total pollen sum through the majority of the late Holocene. Studies have reported similar peat formation, as depicted by the ‘Anderson model’ (Anderson 1964), in Singapore (Taylor *et al*. 2001), and West Kalimantan (Anshari *et al*. 2004) coinciding with the onset of sea-level fall and coastal progradation, recorded *c*. 4000 Cal. years BP in the South China sea (Maloney 1992; Hesp *et al*. 1998; Proske *et al*. 2011). Dommain, Couwenberg & Joosten (2011) documented this process of peat swamp development with sea-level regression across Southeast Asia during the Late Holocene. In the Peat Swamp Fragment site, evidence suggests that the peat swamp started to develop at this time, and in the Deforested Peatland at approximately 1500 Cal. years BP and Converted Peatland at 2800 Cal. years BP, reflecting differing proximities to the coast and inland rivers. (For a more detailed description of landscape development in Converted Peatland, refer to Cole 2013.)

In contrast to the relative dominance and stability of the PSF ecological group through time, within the forest, there has been constant fluctuation between the pioneer and mature PSF communities. The majority of the taxa recorded can be attributed to Anderson's (1964) Phasic Community I, that is, pioneer species and those found on shallow peat at the edge of a dome (Anderson 1964), as might be expected given the relatively short peat cores recovered here (Appendix S3). No evidence was found of successional patterns similar to those documented in peat cores taken from Sarawak by Anderson & Muller (1975). The observed internal dynamism rather represents local regeneration dynamics within the peat swamp forest, for example, gap-phase replacement (Flenley & Butler 2001) associated with natural phenomena such as wind-throw disturbance. Such processes are important for maintaining species diversity and ecosystem functioning (Hector & Bagchi 2007). The only notable changes in the external levels of PSF within the landscape occur in the last 500 years and predominantly in Peat Swamp Fragment and Converted Peatland sites. During this time, degraded peat taxa increase, indicating a conversion of peat swamp forest, most likely related to human land-use change for agricultural production.

### What indicators of past disturbance are there and when?

The lack of coincident evidence of vegetation change with differing ENSO intensity across sites suggests that climatic variability has not acted as a significant form of disturbance in these coastal peat swamp forests. Results of a study synthesizing peat accumulation data from across coastal peat domes in Southeast Asia (Dommain, Couwenberg & Joosten 2011) demonstrate that these ecosystems have shown resilience to falling sea levels and dry El Niño episodes over the late Holocene, supporting the finding that climatic variability in the past has not caused significant disturbance. There also appears to have been limited impact on the peat swamp forest ecosystem or peat substrate during periods of elevated burning or open vegetation (albeit rare) prior to *c*. 500 Cal. years BP, suggesting that the associated potential perturbation factors have not significantly disturbed these peat swamp forests in the past. This is exemplified in particular by the dynamic response of the peat swamp forest in the Peat Swamp Fragment site, via an elevated abundance of PSF pioneers, in the period following the spike in open vegetation *c*. 2200 Cal. years BP, itself likely to be linked to a period of elevated burning and the Arid Tropics event (Fig.3). Here, PSF vegetation demonstrated persistence despite what appeared to be an extended period of exposure to multiple disturbances.

In the last several hundred years, however, the fire and open vegetation proxies do appear to reflect the presence of drivers of disturbance in these peat swamp forest ecosystems. Although fire has been present throughout the past in all sites and in some cases to levels exceeding recent ones (i.e. *c*. 2000–3000 Cal. years BP linked with ENSO-induced drying), it only appears to coincide with inferred forest disturbance in the last *c*. 500 years. Open vegetation follows a similar trend: there is a dramatic increase across all sites within the preceding two centuries. Prior to this, open vegetation was at minimal levels. Despite other studies suggesting that humans have been present and exerting significant impacts on the wet tropical forests of this region since the early Holocene (Flenley 1988; Hunt & Premathilake 2012), or indeed prior to this (Barker *et al*. 2007; Hunt, Gilbertson & Rushworth 2007; Higham *et al*. 2009), results here indicate that a detectable anthropogenic legacy in the coastal peat swamp forests has been a relatively recent phenomenon. There are several lines of evidence in support of this. Firstly, the significant increase in monolete spores in all sites over the last 300 years (a major component of the open vegetation ecological group) may result from large increases in the edible fern *Stenochlaena palustris* (Blechnaceae), locally known as *paku miding*, which grows highly successfully on peat soils where forest has been cleared. Secondly, people reported to have established settlements in these areas only in the recent past (Cole 2013). Thirdly, an extensive study of Sarawak's peatlands performed in the late 1970s ascribed the development of this ecosystem to the last 30 years (Liong & Siong 1979); other studies similarly report of human activities in these peatland ecosystems to have increased rapidly over the last two decades (Miettinen & Liew 2010). In addition, evidence from across Southeast Asian swamps shows increased biomass burning only within the last two to three centuries (Hope, Chokkalingam & Anwar 2005) or even decades (Taylor 2010). Thus, this result, in combination with the recent and simultaneous elevation of both fire and open vegetation levels, suggests a strong association with local anthropogenic activity and further, that anthropogenic forest degradation is likely to have involved biomass burning.

### How did the peat swamp forest vegetation respond to these disturbances?

Answering this question requires an evaluation of the *resilience* of these vegetation communities. In this study, a resilient peat swamp forest is described as one that maintains its function despite experiencing perturbation, manifesting in the persistence and/or regeneration of vegetation common to that ecosystem, that is, types observed during the baseline periods. This appears to be the case throughout periods of increased burning and climatic changes prior to *c*. 500 years ago, with peat swamp forest dominating, suggesting that none of these apparently *natural* disturbances have challenged the resilience of these coastal ecosystems. Although the disturbance indicators cannot be separated such that their individual effects can be assessed, results do suggest that the higher intensities and combinations of the different drivers in the last two centuries may have impacted more significantly on the peat swamp forest. For example, the inferred presence of elevated local burning and human impact (via fossil charcoal and open vegetation proxies respectively) from *c*. 500 Cal. years BP (coincident with the period when people are thought to have started clearing these ecosystems) do not appear to correspond with peat swamp forest regeneration: a key process in a functioning forest. Assessing whether a threshold has been crossed here and thus resilience compromised, is hindered by a lack of data. However, evidence of long-term stability of PSF vegetation in these sites prior to these recent impacts, coupled with that of internal dynamism between mature and pioneer taxa, suggest no thresholds were approached and the ecosystem demonstrated resilience throughout the period pre-*c*. 500 Cal. years BP.

Another indicator that the resilience of the peat swamp forest is compromised under elevated levels and combinations of disturbance after this point is the greater degree of fluctuations of the PSF ecological groups. Work by Carpenter & Brock (2006) and Dakos *et al*. (2012), for example, equates increasing fluctuation in ecological components to ecosystem instability. Wösten *et al*. (2008) report that whilst intact peat swamp forests demonstrate resilience to disturbances to their hydrological integrity, in a degraded state, these ecosystems are more susceptible to further disturbance, especially fire. Similarly, Nishimura *et al*. (2007) suggest that peat swamp forests have the potential to recover from a single drought event, but not a succession of them. Whether there is a measurable critical threshold for these coastal peat swamp forests, for instance hydrological integrity (Dommain, Couwenberg & Joosten 2011), whether other factors also contribute to determining ecosystem resilience or whether such a potential threshold is being surpassed in these sites require further investigation.

### Limitations of tropical palaeoecology

The palaeoecological approach adopted here offers new insights into the long-term ecological functioning, vegetation baselines and effects of environmental drivers on these peat swamp forests that cannot be obtained through field studies, on which much of today's ecological theory is based. However, it is important to understand the limitations of the discipline and, in particular, the nuances of interpreting pollen data from tropical forested and non-forested environments. One notable factor is that pollen grains and spores produced by plants characteristic of open areas are generally anemophilous, that is, wind-transported, and as such, these plants have evolved to produce large volumes of highly mobile pollen grains/spores. This mobility enables transport over longer distances by wind and thus these pollen grains/spores can provide a signal for regional vegetation. However, the zoophilous, that is, insect-transported pollen produced by most tropical forest trees (Colinvaux & De Oliveira 2001) give a more local signal, and large quantities can accumulate in one site distorting pollen-based vegetation reconstructions. In general, tropical peat swamp forests comprise a dense closed-canopy environment, which can restrict long-distance pollen and charcoal transportation to the coring sites (Muller 1963). Therefore, in combination with the form of pollen grain transport occurring, high concentrations of a certain pollen grain may not reflect the regional dominance of that taxon, simply a high local deposition obscuring wider landscape change (Haseldonckx 1977). Studies performed in other dense tropical forests claim that pollen grains found in sediment cores are likely to have been generated by parent plants within a distance of only 20–50 m from the site (Jolly *et al*. 1996; Elenga *et al*. 2000). However, more studies are needed in this specific ecological context in order to improve the objectivity and thus accuracy of interpretations of past plant communities (for regional examples see Kershaw & Strickland 1990 and Taylor *et al*. 2001). In this study, noteworthy changes in fossil pollen coincident with disturbance events, used to assess vegetation responses and thus forest resilience, may not be apparent as a result of the lifespan of rainforest trees, covering typically two centuries (Chambers, Higuchi & Schimel 1998): standing trees can continue to produce pollen despite a broad loss of surrounding forest and failure of forest regeneration. Another important consideration is the temporal resolution of sampling points; fundamental to making ecologically viable interpretations of the fossil pollen data. In addition, acknowledgement of the inherent ranges of radiocarbon dates used in the construction of chronologies must underpin analysis and attempts at deciphering the timing, and indeed magnitude and relative impact of different disturbances. In a similar study, Anshari *et al*. (2004) suggest that peat development and its attributes have had a complex relationship with climatic changes and human activity during the Holocene, making the allocation of regional environmental drivers, especially climatic impacts, challenging. Climatic variability predominantly manifests in other environmental changes, that is, indirect drivers of ecosystem change via fire or drying, further complicating the characterization of individual disturbance events.

### Management considerations

Results from this study provide insights into how these peat swamp forests can be managed to foster ecosystem persistence in the face of contemporary and future change. Although an ensuing loss of resilience in these studied sites cannot be inferred from the available data, the elevations in disturbance indicators observed in the recent past provide a warning signal for potential future ecological shifts, in line with the recent inferred declines in PSF vegetation within the Peat Swamp Fragment and Converted Peatland sites. The impacts these forests are facing today are of a higher magnitude and novel type to those experienced in the past, for example, the contemporary logging, subsequent drainage and establishment of oil palm plantations over vast areas. To date, there is no evidence to suggest that these forests are resilient to such disturbance, potentially driving the ecosystems into a landscape trap (Lindenmayer *et al*. 2011). The peat swamp forest itself may maintain its ability to regenerate, but current environmental conditions and land-use practices, with a constant disruption of the soil and local seed sources, limited time between anthropogenic disturbance cycles relative to natural forest recovery times (Cole, Bhagwat & Willis 2014) and increased prevalence and intensity of fires (Hope, Chokkalingam & Anwar 2005), are warranting it a non-renewable resource (Gomez-Pompa, Vasque-Yanes & Guevara 1972). A recent study of Southeast Asia peatlands predicted that if their current rate of deforestation is maintained, these forests will have disappeared by 2030 (Miettinen *et al*. 2012). If halted however, and hydraulic integrity preserved (Dommain, Couwenberg & Joosten 2010), forests can recover with little or no assistance, as exemplified by the rapid regeneration observed in a deforested and partially drained peat swamp in Kuching, Sarawak, where mowing is required to prevent forest regrowth (personal observation, L.E.S.C.). Finally, there is limited evidence of successful restoration in heavily degraded peatlands (van Eijk *et al*. 2009; Jaenicke, Englhart & Siegert 2011; Graham & Page 2012), such as the Ex-Mega Rice Project area in Central Kalimantan (Page *et al*. 2009).

Sustainable Forest Management (SFM) (Imai *et al*. 2009) and paludiculture, the practice of wetland agriculture (FAO 2012), are being explored as a means of delivering a direct service from these areas without challenging their resilience. However, more research and trialling are required to understand the parameters and potential impacts of these two management strategies before they should be considered for widespread implementation.

Evidence from this study suggests that peat swamp forests are able to respond dynamically to disturbances in the past, but that this resilience may be brought to question when contemporary, human-induced perturbation is introduced. Therefore, designing peatland-use strategies that limit disturbances to natural levels and adopting the precautionary principle where ecological knowledge is still lacking may be central to managing them more sustainably. Whether such an approach can be upheld amongst the contemporary pressures from agricultural markets (Carter *et al*. 2007; Koh & Wilcove 2007) and trends in land use across Southeast Asia (Koh *et al*. 2011; Miettinen, Shi & Liew 2011, 2012) is questionable. This study provides key insights into the long-term ecological dynamics, disturbance history and resilience of these tropical ecosystems, providing information important for the debate on sustainable peat swamp forest management in the face of contemporary and future disturbance.

## Data accessibility

Taxanomic data, i.e. fossil pollen and spore types, and micro- and macrocharcoal counts displayed in Figs 2 and 3, are available on DRYAD – entry doi: 10.5061/dryad.b5r07.

## Supporting Information

**Appendix S1.** Details of fossil pollen sampling frequency in each sediment core.

**Appendix S2.** Photographic images of common fossil pollen and spore taxa.

**Appendix S3.** Age-depth profiles of each sediment core. **Figure S1.** Age-depth profile of each sediment core: (a) Deforested Peatland, (b) Peat Swamp Fragment, and (c) Converted Peatland.

**Table S1.** ENSO-related Late Holocene climatic changes in the region surrounding northern Borneo (with associated references).

**Table S2.** Ecological groupings of plant taxa identified through fossil pollen analysis.
