# Supplementary material for: Long-term disturbance dynamics and resilience of tropical peat swamp forests
Source: J Ecol. 2015 Jan 7;103(1):16–30. doi: 10.1111/1365-2745.12329 (PMC4477911; doi:10.1111/1365-2745.12329)
Supplement: Supplementary file 1 — Appendix S1. Details of fossil pollen sampling frequency in each sediment core. [file jec0103-0016-sd1.pdf]

Appendix S1 Details of fossil pollen sampling frequency in each sedimentary core.

Pollen preparations were made from 1 cm^3 samples taken at intervals of 8 cm throughout the length of the Deforested Peatland core; at 4 cm intervals for the majority of the Peat Swamp Fragment core, and 6 cm and 8 cm intervals towards the top; and at 2 cm intervals for the majority of the Converted Peatland core, and 4 cm, 8 cm and 16 cm intervals towards the base, to minimise the difference of time intervals between samples within and across cores.
