# Supplementary material for: Long-term disturbance dynamics and resilience of tropical peat swamp forests
Source: J Ecol. 2015 Jan 7;103(1):16–30. doi: 10.1111/1365-2745.12329 (PMC4477911; doi:10.1111/1365-2745.12329)
Supplement: Supplementary file 2 — Appendix S2. Photographic images of common fossil pollen and spore taxa. [file jec0103-0016-sd2.pdf]

Appendix S2: Photographic Images of Common Fossil Pollen & Spore Taxa

List of taxa represented in photographs of pollen grains and spores, ordered alphabetically in ecological groups (Table 2):

1. Lycopodium (*marker spore*)

PEAT SWAMP FOREST (PSF)

2. Alangiaceae *Alangium*
3. Anacardiaceae type
4. Anisophyllaceae/Rhizophoraceae *Combretocarpus*
5. Apocynaceae type
6. Aquifoliaceae *Ilex*
7. Araceae
8. Arecaceae *Calamus*
9. Casuarinaceae *Casuarina*
10. Crypteroniaceae *Dactylocladus* (a & b)
11. Dipterocarpaceae Menispermaceae type
12. Dipterocarpaceae type
13. Ebenaceae *Diospyros* (a & b)
14. Euphorbiaceae *Blumeodendron*
15. Fabaceae *Copaifera* (a & b)
16. Fabaceae *Koompassia* sim
17. Fagaceae *Castanopsis*
18. Guttiferae *Garcinia*
19. (Icacinaceae – former family pre-reclassification) Stemonuraceae *Stemonurus*
20. Loranthaceae
21. Meliaceae *Aglaia*
22. Pandanaceae *Pandanus*
23. Rhizophoraceae *Rhizophora*
24. Rubiaceae *Uncaria*
25. Sapotaceae *Palaquium*
26. Sapotaceae type (a & b)
27. Theaceae *Eurya* (a & b)
28. Thymeliaceae *Gonystulus*

PEAT SWAMP FOREST – PIONEERS (PSF+)

29. Elaeocarpaceae *Elaeocarpus*
30. Euphorbiaceae *Macaranga* (a & b)
31. Euphorbiaceae *Mallotus*
32. Moraceae *Ficus*
33. Myrsinaceae type (a & b)
34. Myrtaceae *Syzygium*
35. Piperaceae *Piper*
36. Ulmaceae *Trema*

DEGRADED PEAT (DP)

37. Asteraceae
38. Asteraceae type
39. Dilleniaceae *Dillenia* (a & b)
40. Melastomataceae *Melastoma*
41. Urticaceae *Poikilospermum*

OTHER FOREST (OF)

42. Ericaceae *Rhododendron*
43. Rosaceae type (a & b)
44. Symplocaceae type

COASTAL VEGETATION (CV)

45. Arecaceae *Cyrtostachys* (a & b)
46. Arecaceae *Oncosperma*
47. Ochnaceae *Brackenridgea* sim
48. Rhizophoraceae *Ceriop*s sim
49. Simaroubiaceae *Quassia*
50. Sonneratiaceae *Sonneratia*

OPEN VEGETATION

51. Cyperaceae (a & b)
52. Monolete (a & b)
53. Poaceae (a & b)
54. Trilete (a & b)

The scale bar (▬) represents *c*. 10μm, giving an indication of the size of each grain and spore.

First developed by Benninghoff and Kapp (1962), the following system of notation has been used to reflect the level of certainty in fossil pollen identifications made: ‘comp’ indicates a grain that is almost certainly the same as the reference taxon; ‘sim’, one that is more similar to the reference taxon than any other known reference taxa, but there is less certainty in the association; and ‘type’, a grain corresponds with one morphology within a polymorphic taxonomic unit.

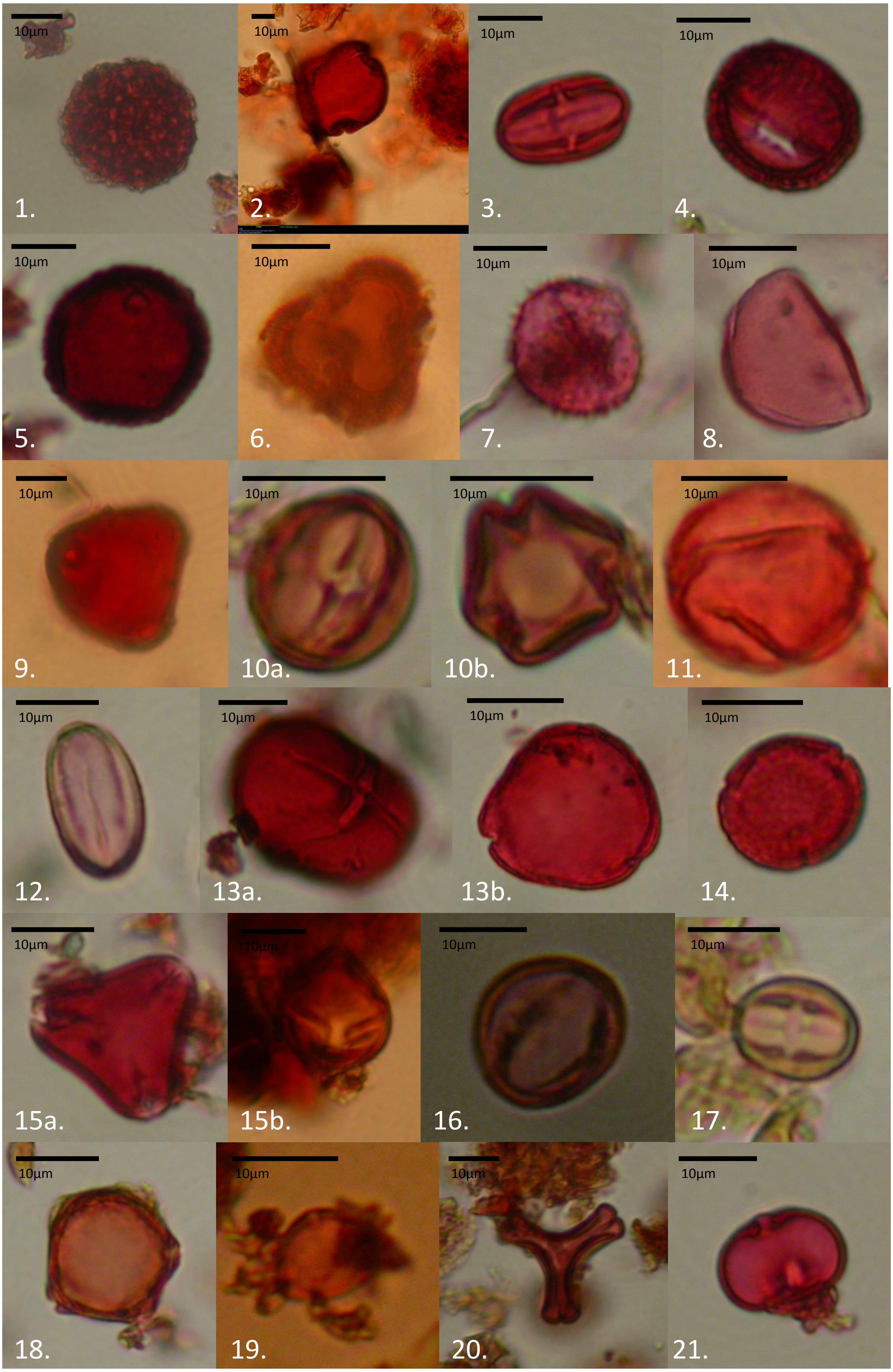
10µm
1.
10µm
2.
10µm
3.
10µm
4.
10µm
5.
10µm
6.
10µm
7.
10µm
8.
10µm
9.
10µm
10a.
10µm
10b.
10µm
11.
10µm
12.
10µm
13a.
10µm
13b.
10µm
14.
10µm
15a.
10µm
15b.
10µm
16.
10µm
17.
10µm
18.
10µm
19.
10µm
20.
10µm
21.

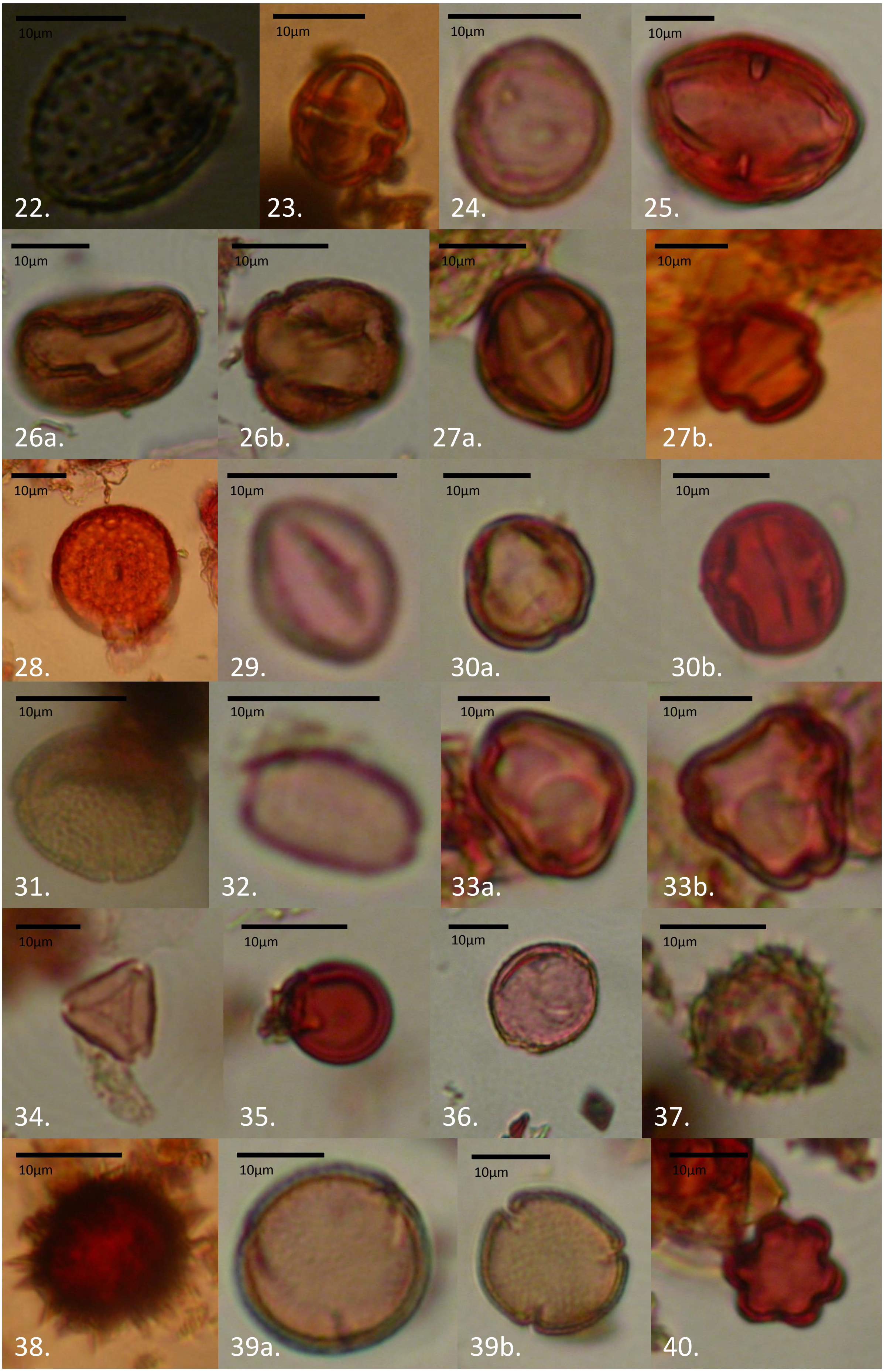
10µm
22.
23.
24.
25.
26a.
26b.
27a.
27b.
28.
29.
30a.
30b.
31.
32.
33a.
33b.
34.
35.
36.
37.
38.
39a.
39b.
40.

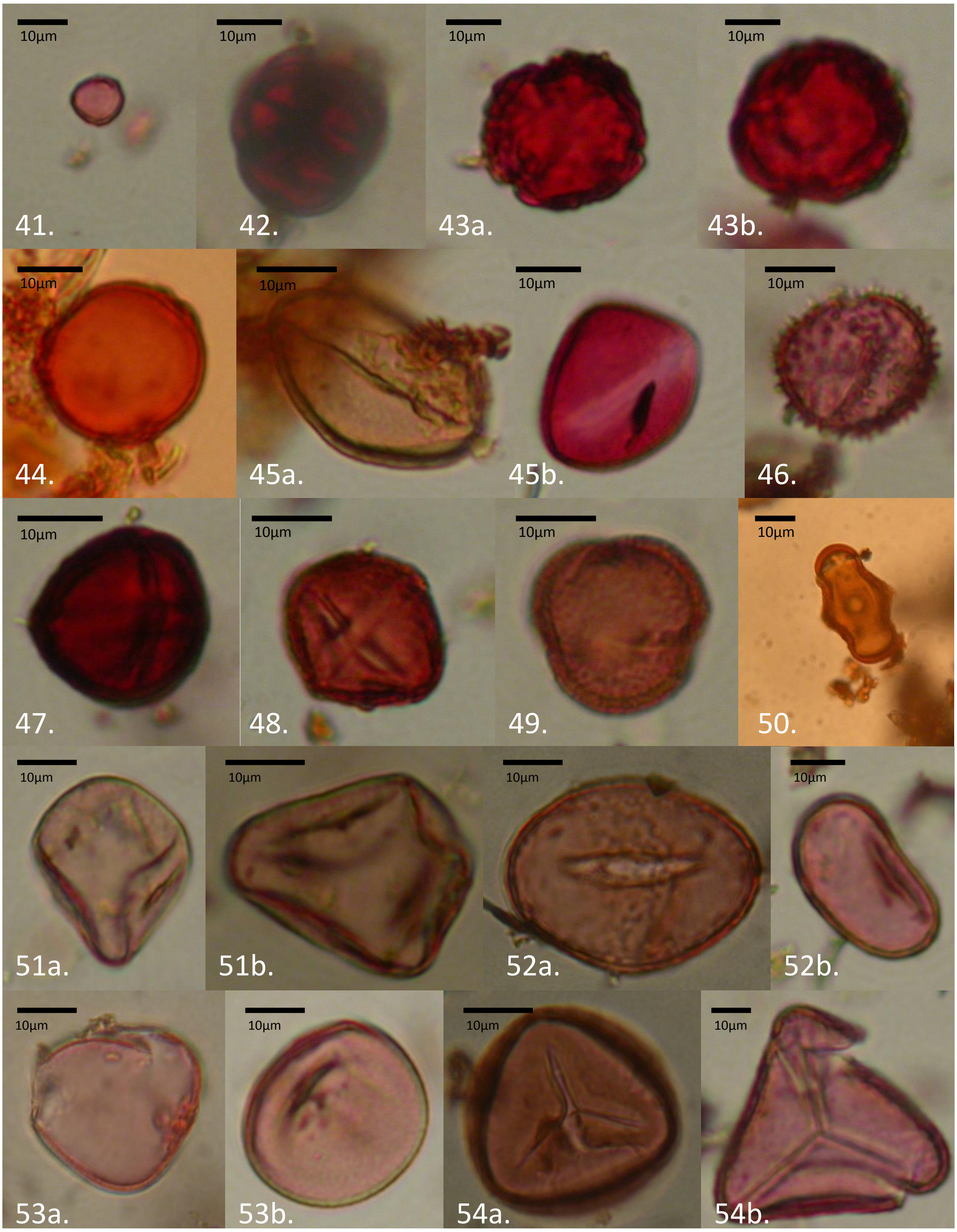
41.
42.
43a.
43b.
44.
45a.
45b.
46.
47.
48.
49.
50.
51a.
51b.
52a.
52b.
53a.
53b.
54a.
54b.
10µm
