# Supplementary material for: Long-term disturbance dynamics and resilience of tropical peat swamp forests
Source: J Ecol. 2015 Jan 7;103(1):16–30. doi: 10.1111/1365-2745.12329 (PMC4477911; doi:10.1111/1365-2745.12329)
Supplement: Supplementary file 3 [file jec0103-0016-sd3.pdf]

**Appendix S3** Age-depth profiles of each sediment core.

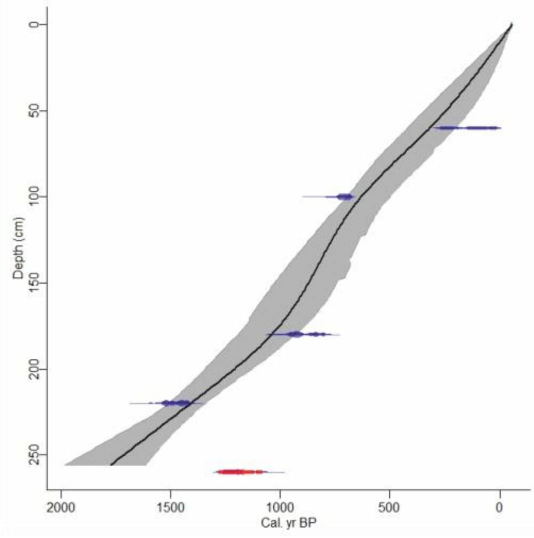


(a)

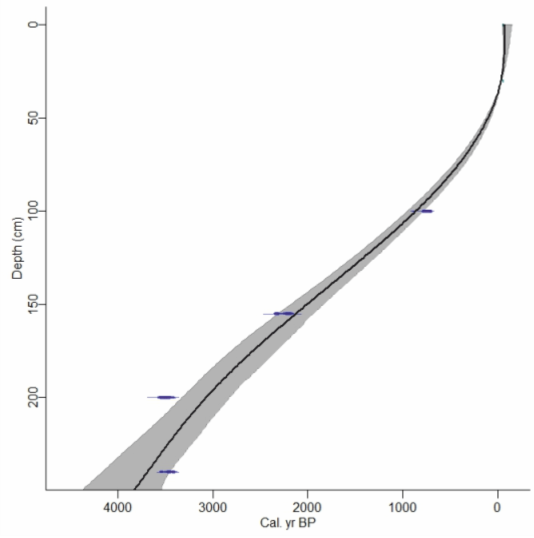


(b)

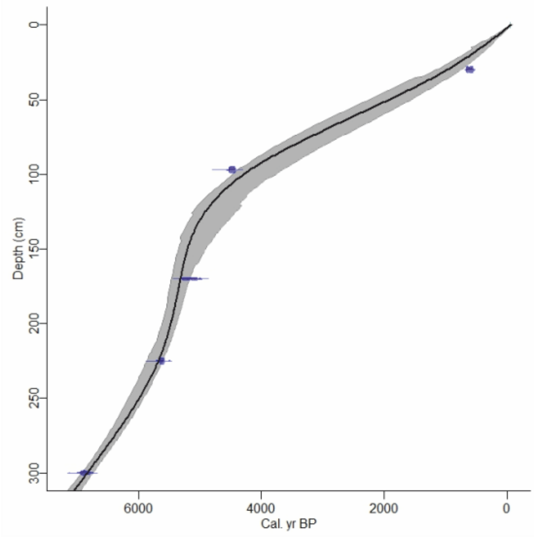


(c)

**Fig. S1.** Age-depth profile of each sedimentary core: (a) Deforested Peatland, (b) Peat Swamp Fragment, and (c) Converted Peatland. A smoothing spline model provided the best-fit across the radiocarbon dates obtained for each core (five per site; Table 2). The calibrated age for the base of each core was extrapolated using the model, and for the surface through setting the sample at 0cm to -55 years (i.e. the approximate time since the impact of above-ground nuclear testing and the fossil fuel effect were identified (Bowman 1990)). The distributions of calibrated ages per sediment sample (dated via radiocarbon techniques) are

shown in blue. (The basal date in the Deforested Peatland site (outlier shown in red), estimated from a sample taken at 260cm depth, represents an age inversion and thus has not been used in developing the age-depth model.)
