# Supplementary material for: Long-term disturbance dynamics and resilience of tropical peat swamp forests
Source: J Ecol. 2015 Jan 7;103(1):16–30. doi: 10.1111/1365-2745.12329 (PMC4477911; doi:10.1111/1365-2745.12329)
Supplement: Supplementary file 4 — Table S1. ENSO-related Late Holocene climatic changes in the region surrounding northern Borneo (with associated references). [file jec0103-0016-sd4.pdf]

Table S1 Major periods of ENSO-related climatic change, the score for relative intensity awarded to each and references used to construct them.

ENSO Time Period (Cal. years BP)	ENSO-related characteristics of period	Score	References (in order of characteristics)
0 – 100	Intensified ENSO	2	Gagan *et al.* (2004)
100 – 600	Little Ice Age; declining ENSO frequency	1	Grieβinger *et al.* (2011); Moy *et al.* (2002)
600 – 1700	Amplification of ENSO	3	Woodroffe *et al.* (2003)
1700 – 3000	Abrupt increase in ENSO magnitude & Holocene maximum	2	Gagan *et al.* (2004); Woodroffe *et al.* (2003)
3000 - 3800	Reduced ENSO intensity	1	Woodroffe *et al.* (2003)
3800 - 5000	Onset of modern ENSO variability; reductions in Summer East Asia Monsoon; series of periods of tropical aridity; drought in New Guinea	2	Gagan *et al.* (2004); Selvaraj *et al.* (2011); Mayewski *et al.* (2004); Haberle *et al.* (2001)
5000 - 7000+	Relatively high precipitation in northern Borneo, & a relatively stable climate regionally; Summer Monsoon dominant & ENSO weak	0	Partin *et al.* (2007); Haberle *et al.* (2001); Abram *et al.* (2007); Moy *et al.* (2002)

References

Abram, N.J., Gagan, M.K., Liu, Z., Hantoro, W.S., McCulloch, M.T. & Suwargadi, B.W. (2007) Seasonal characteristics of the Indian Ocean Dipole during the Holocene epoch. *Nature*, **445**, 299–302.

Gagan, M.K., Hendy, E.J., Haberle, S.G. & Hantoro, W.S. (2004) Post-glacial evolution of the Indo-Pacific Warm Pool and El Niño-Southern oscillation. *Quaternary International*, **118-119**, 127–143.

Grieβinger, J., Bräuning, A., Helle, G. & Schleser, G. (2011) Late Holocene Asian summer monsoon variability reflected by δ18O in tree-rings from Tibetan junipers. *Geophysical Research Letters*, **38**, L03701. doi:10.1029/2010GL045988

Haberle, S.G., Hope, G.S. & Van Der Kaars, S. (2001) Biomass burning in Indonesia and Papua New Guinea: Natural and human induced fire events in the fossil record. *Palaeogeography, Palaeoclimatology, Palaeoecology*, **171**, 259–268.

Mayewski, P.A., Rohling, E.E., Stager, J.C., *et al.* (2004) Holocene climate variability. *Quaternary Research*, **62**, 243–255.

Moy, C.M., Seltzer, G.O., Rodbell, D.T., & Anderson, D.M. (2002) Variability of El Nino/Southern Oscillation activity at millennial timescales during the Holocene epoch. *Nature,* **420**, 162-165.

Partin, J.W., Cobb, K.M., Adkins, J.F., Clark, B. & Fernandez, D.P. (2007) Millennial-scale trends in west Pacific warm pool hydrology since the Last Glacial Maximum. *Nature*, **449**, 452–455.

Selvaraj, K., Arthur Chen, C-T., Lou, J-Y. & Kotlia, B.S. (2011) Holocene weak summer East Asian monsoon intervals in Taiwan and plausible mechanisms. *Quaternary International*, **229**, 57–66.

Woodroffe, C.D., Beech, M.R. & Gagan, M.K. (2003) Mid-late Holocene El Niño variability in the equatorial Pacific from coral microatolls. *Geophysical Research Letters*, **30**, 10–11.
