# Supplementary material for: Long-term disturbance dynamics and resilience of tropical peat swamp forests
Source: J Ecol. 2015 Jan 7;103(1):16–30. doi: 10.1111/1365-2745.12329 (PMC4477911; doi:10.1111/1365-2745.12329)
Supplement: Supplementary file 5 — Table S2. Ecological groupings of plant taxa identified through fossil pollen analysis. [file jec0103-0016-sd5.pdf]

Table S2 Ecological groupings of plant taxa identified through fossil pollen analysis, showing the complete list of pollen grains and spores counted and used for past vegetation reconstruction, along with the descriptions of the key characteristics of each group.

**Plant family**	***Pollen taxon***	**Plant family**	***Pollen taxon***
PEAT SWAMP FOREST (PSF) - if taxa present in peat swamp forest, assumed to be an old-growth forest			
Araceae		Hammelidaceae	*Altingia*
Arecaceae	*Calamus*	Icacinaceae	Icacinaceae type
	Cyrtosperma	Lamiaceae	*Salvia*
	Arecaceae type	Lauraceae	*Litsea*
Alangiaceae	*Alangium*	Loganiaceae	*Fagraea*
Anacardiaceae	*Campnosperma* sim		*Fagraea* sim
	Melanorrhoea comp	Loranthaceae	Loranthaceae type
Anacardiaceae	Anacardiaceae type	Meliaceae	*Aglaia* sim
Anisophyllaceae/ Rhizophoraceae	*Combretocarpus*		*Trichilia* sim
	Combretocarpus sim	Moraceae	*Artocarpus*
	4-colporate	Myristicaceae	Myristicaceae type
	Anisophyllaceae type	Myrsinaceae	*Rapanea*
Apocynaceae	Apocynaceae type	Orchidaceae	
Aquifoliaceae	*Ilex*	Pandanaceae	*Pandanus*
Araliaceae	*Schefflera* sim	Podocarpaceae	*Dacrydium*
	Rutaceae type	Polygalaceae	Polygalaceae type
	Araliaceae type	Rhamnaceae	Rhamnaceae type
Burseraceae	Burseraceae type	Rhizophoraceae	Rhizophoraceae type
Casuarinaceae	*Casuarina*	Rosaceae	*Parastemon*
Celastraceae	*Lophopetalum*		Rosaceae type
	Celastraceae type		Rosaceae/Sterculiaceae type
Crypteroniaceae	*Dactylocladus*	Rubiaceae	*Gardenia*
Cunoniaceae	*Weinmannia*		*Ixora*
Dipterocarpaceae	*Shorea* sim		*Ixora* type
	Dipterocarpaceae type		*Nauclea*
	Dipterocarpaceae Fagaceae type		*Nauclea* sim
	Dipterocarpaceae Menispermaceae type		*Tarenna* sim
Ebenaceae	*Diospyrsos*		*Thysanospermum*
	Diospyrsos sim		*Timonius*
	Ebenaceae Fagaceae *Diospyrsos* type		*Timonius* sim
Euphorbiaceae	*Blumeodendron*		*Uncaria*
	Cephalomappa sim	Rutaceae	*Melicope*
	Glochidion		*Tetractomia* sim
	Euphorbiaceae type	Sapotaceae	*Palaquium*
Fabaceae	*Albizia* comp		*Palaquium* comp
	Archidendron		*Planchonella* comp
	Copaifera		Sapotaceae type
	Copaifera comp	Stemonuraceae	*Stemonurus*
	Koompassia	Sterculiaceae	*Reevesia*
	Koompassia sim		Sterculiaceae type

**Plant family**	***Pollen taxon***	**Plant family**	***Pollen taxon***
Fagaceae	*Castanopsis*	Theaceae	*Eurya*
	Fagaceae type		Theaceae type
Flacourtiaceae	*Casearia*	Thymeliaceae	*Gonystylus*
	Casearia sim	Tiliaceae	Tiliaceae type
Guttiferae	*Callophylum*	Trigoniaceae	*Trigoniastrum*
	Callophylum sim		Trigoniaceae type
	Cratoxylon sim		
	Garcinia		
	Guttiferae Cratoxylon		
	Theaceae type		

PEAT SWAMP FOREST pioneers (PSF+) - if taxon increases in abundance in pollen diagram, indicates early successional plant community of secondary peat swamp forest

Acanthaceae		Menispermaceae	*Fibraurea*
Annonaceae	Annonaceae type		*Fibraurea* sim
Elaeocarpaceae	*Elaeocarpus*	Moraceae	*Ficus*
	Elaeocarpus sim	Myearstaceae	*Syzygium*
	Elaeocarpaceae type		Myrtaceae type
Euphorbiaceae	*Baccaurea*	Piperaceae	*Piper*
	Baccaurea comp	Sapindaceae	*Dodonea*
	Macaranga		*Pometia* comp
	Macaranga sim		Sapindaceae type
	Mallotus	Ulmaceae	*Trema*
	Mallotus type	Verbenaceae	Verbenaceae type
		Vitaceae	*Cayratia*

DEGRADED PEAT (DP) - taxa not found in older-growth peat swamp forest or found in greater abundance in disturbed areas of peat where the forest canopy is open

Asteraceae	Asteraceae type	Lecythidaceae	*Barringtonia* type
Celastraceae	*Bhesa* sim	Melastomataceae	*Melastoma*
Dilleniaceae	*Dillenia*	Rutaceae	*Euodia*
	Dillenia sim		*Euodia* sim
Escallionaceae	*Polyosma* sim	Smilacaceae	*Smilax*
Fabaceae	*Uraria*	Urticaceae	*Poikilospermum*
	Uraria sim		

Plant family	*Pollen taxon*	Plant family	*Pollen taxon*
OTHER FOREST (OF) - other forest (non-peat swamp forest taxa), e.g. swamp forest or forest on mineral soils			
Asclepidiaceae	*Dischidia*	Myrsinaceae	*Ardisia* sim
Bombacaceae	*Durian* comp	Rosaceae	*Rubus*
	Bombacaceae type		*Rubus* sim
Combretaceae			Rosaceae type
Cycadaceae	*Cycas*	Symplocaceae	*Symplocos*
Ericaceae	*Rhododendron*		*Symplocos* type
Icacinaceae	*Platea*	Theaceae	*Eurya* sim
	Icacinaceae type	Vitaceae	*Ampelocissus* sim
	Juglandaceae Myrtaceae type		
COASTAL VEGETATION (CV) - coastal vegetation associated with succession to peat from mangrove/littoral habitat type			
Acanthaceae	Acanthaceae type	Pteridaceae	*Acrostichum*
Arecaceae	*Cyrtostachys*	Rhizophoraceae	*Ceriops* sim
	Oncosperma		*Rhizophora*
	Oncosperma sim		*Rhizophora* type
Avicenniaceae	*Avicennia*	Simaroubiaceae	*Quassia*
Combretaceae	*Lumnitzera*		*Quassia* sim
Malvaceae	*Hibiscus*	Sonneratiaceae	*Sonneratia*
	Malvaceae type		
Ochnaceae	*Brackenridgea*		
	Brackenridgea sim		
	Ochnaceae type		
DISTURBANCE TAXA - disturbance tolerant vegetation indicative of open environments (not included in pollen sum)			
Poaceae		Lygodiaceae	
Cyperaceae		Cyathaceae	
Monolete		Trilete	
Lycopodiaceae	*Lycopodium cernuum*		
	Lycopodium phlegmaria		
